# Medical-informed machine learning: integrating prior knowledge into medical decision systems

**DOI:** 10.1186/s12911-024-02582-4

**Published:** 2024-06-28

**Authors:** Christel Sirocchi, Alessandro Bogliolo, Sara Montagna

**Affiliations:** Department of Pure and Applied Sciences, University of Urbino, Piazza della Repubblica, 13, Urbino, 61029 Italy

**Keywords:** Machine learning, Domain knowledge, Rule-based, Integration, Diabetes

## Abstract

**Background:**

Clinical medicine offers a promising arena for applying Machine Learning (ML) models. However, despite numerous studies employing ML in medical data analysis, only a fraction have impacted clinical care. This article underscores the importance of utilising ML in medical data analysis, recognising that ML alone may not adequately capture the full complexity of clinical data, thereby advocating for the integration of medical domain knowledge in ML.

**Methods:**

The study conducts a comprehensive review of prior efforts in integrating medical knowledge into ML and maps these integration strategies onto the phases of the ML pipeline, encompassing data pre-processing, feature engineering, model training, and output evaluation. The study further explores the significance and impact of such integration through a case study on diabetes prediction. Here, clinical knowledge, encompassing rules, causal networks, intervals, and formulas, is integrated at each stage of the ML pipeline, resulting in a spectrum of integrated models.

**Results:**

The findings highlight the benefits of integration in terms of accuracy, interpretability, data efficiency, and adherence to clinical guidelines. In several cases, integrated models outperformed purely data-driven approaches, underscoring the potential for domain knowledge to enhance ML models through improved generalisation. In other cases, the integration was instrumental in enhancing model interpretability and ensuring conformity with established clinical guidelines. Notably, knowledge integration also proved effective in maintaining performance under limited data scenarios.

**Conclusions:**

By illustrating various integration strategies through a clinical case study, this work provides guidance to inspire and facilitate future integration efforts. Furthermore, the study identifies the need to refine domain knowledge representation and fine-tune its contribution to the ML model as the two main challenges to integration and aims to stimulate further research in this direction.

## Introduction

Machine learning (ML) has revolutionised various industries, from manufacturing to governance, and is now making its way into healthcare - a sector traditionally resistant to technological disruptions. ML has achieved human-level performance in various domains of clinical medicine, spanning from oncology [1] and orthopaedics [2] to ophthalmology [3] and general practice, and has been shown to predict hospitalisation duration [4], reduce waiting times [5], improve medication adherence [6], customise medication dosages [7], among others. Notably, these models outperformed human physicians in some cases, leading to the development of computer-aided diagnosis systems [8]. However, while some of these systems have been FDA-approved for healthcare use, they were primarily developed within radiology and cardiovascular specialities [9, 10], followed by neurology and haematology [11]. Ensuring effective deployment of ML models in clinical settings requires not only demonstrating high prediction accuracy during training but also their actual impact on clinical outcomes [12]. To this day, thousands of studies have applied ML algorithms to medical data, but only a handful have significantly contributed to clinical care. This lack of impact contrasts sharply with the significant relevance of ML in other industries [13].

The article emphasises the importance of ML in medical data analysis but acknowledges that ML alone may not capture the full complexity of clinical data due to limited data availability. The authors argue that integrating medical domain knowledge throughout the ML pipeline should become a standard practice in the medical field. This integration is essential to build predictive models with qualities that are particularly desirable in the healthcare sector, thereby facilitating their adoption in clinical practice. Such models must not only attain high accuracy with limited data but also provide good explanations and adhere to current guidelines in order to foster trust in clinicians and guarantee continuity of care.

The article examines past efforts to integrate prior medical knowledge into clinical research, noting that the medical community has begun to recognise this need for integration but that the approaches thus far have been scattered and focused on particular medical data types. Then, the article presents a structured overview of existing strategies, mapping them onto the corresponding ML phases (i.e. data pre-processing, feature engineering, model learning, and output evaluation) and providing general guidelines for integrating prior knowledge at each phase.

The study further explores the significance and impact of such integration through a case study on diabetes prediction. Domain knowledge formalised as rules, causal networks, intervals, and formulas, was integrated at each stage of the ML pipeline, resulting in a range of hybrid models. The results underscore the advantages of this integration in terms of accuracy, interpretability, data efficiency, and compliance with clinical guidelines. The integrated models often outperformed purely data-driven methods, highlighting how domain knowledge can enhance ML models by improving model generalisation. In certain cases, this integration improved model interpretability and alignment with established clinical guidelines. Notably, tests conducted on subsets drawn from the original dataset demonstrated that integrating knowledge effectively maintains performance in scenarios with limited data.

Finally, the study identifies the need to refine the representation of medical domain knowledge and fine-tune its contribution to the ML model as the two main challenges to integration and critical areas for future research.

## Previous work

The section presents the potential benefits and inherent challenges of ML in clinical settings. It specifically addresses the limitations of fully data-driven models in terms of data requirements, interpretability, accuracy, and alignment with existing medical knowledge, aspects that are critical to the healthcare domain. The section extends to explore the types of domain knowledge prevalent in the medical field and examines previous efforts to incorporate this knowledge into ML models in healthcare.

### Limitations of ML in medicine

Medical data are rapidly expanding with the development of new therapies and diagnostics enabled by advances in immunology and genetics. Health records also accumulate as patients age, develop comorbidities, and undergo more diagnostic testing. Traditional techniques are not equipped to manage this exponential information growth. In contrast, ML algorithms are ideally suited to integrate abundant and heterogeneous data and may be the most feasible option available in many biomedical settings [8]. Moreover, medical decision-making has become increasingly complex outpacing the capacity of the human mind and can no longer be effectively captured by simple, human-readable models [14]. ML, on the other hand, can support medical decision-making in complex scenarios, as it works best when the underlying model arises from non-additivity and complex interactions between features. However, ML application to clinical settings presents a number of issues, fore and foremost concerning the quantity, quality, and composition of clinical data.

**Data efficiency**. As data for a single patient accumulate, gathering unbiased data across thousands of patients and independent cohorts on the same informative features remains challenging, requiring considerable time, financial resources, specialised instrumentation, and trained personnel [13]. ML models are sensitive to noise and prone to over-fitting when the data is limited or not representative of the population. Therefore, the efficacy of many ML models is contingent upon a large number of samples and an extensive set of features to learn relationships solely from data, a condition rarely met in healthcare contexts [15]. Leveraging existing data sources becomes crucial when collecting large, diverse, and high-quality datasets is not attainable and has contributed significantly to the success of ML across various sectors. However, in clinical research, sharing methods is not common, and access to electronic medical records or clinical registries is limited by data protection policies [16]. Even when sufficient data is available, data classes are often unbalanced complicating disease identification, as most ML models struggle to classify the underrepresented class [8]. Normalisation approaches, such as oversampling the least frequent class, can balance datasets, but the effectiveness of these methods depends on the dataset characteristics [17].

**Accuracy**. Complex dependencies of the pathogenetic mechanisms manifest as inter-patient variability in the form of incomplete (partial presentation of symptoms), imprecise (less specific symptoms), and noisy (unrelated symptoms) data, further complicating modelling efforts. This combination of complex underlying relationships and insufficient data contributes to the current under-performance of ML models, preventing their pervasive adoption and application. Even when proven more accurate than clinicians on average, ML models are unlikely to be approved for clinical practice without high accuracy as errors in healthcare result in enormous costs.

**Interpretability**. In modelling complex clinical problems, there is a tendency to employ more complex ML architectures, which often come at the expense of interpretability. Accurate models are still less trusted and valued by clinicians if they cannot explain their predictions, while interpretable models that share some insight into their decision-making process are more helpful to clinicians as a second opinion [8]. Hence, as ML models grow increasingly complex, interpretability emerges as a crucial factor in advancing the use of ML in the conservative field of medicine. To meet this demand, eXplainable Artificial Intelligence (XAI) has emerged to provide methods that enable human users to understand the outputs generated by ML models [18]. Nonetheless, challenges persist as XAI generally offers post-hoc explainability rather than interpretability by design, which is preferable in clinical applications.

**Coherence**. Traditional ML models frequently lack awareness of the intrinsic structure between attributes, leading to decisions based on confounding variables, improper relationships, or latent variables without physical interpretation [19]. Additionally, ML models often disregard established medical protocols derived from centuries of research and are highly effective in many scenarios. Even when ML models achieve high accuracy and outperform that of existing clinical guidelines, the lack of adherence to the guidelines poses serious concerns, as no error, however rare, would be ethically acceptable if there were a known rule capable of preventing it. Models that are more accurate than the current protocol but fail to correctly predict cases effectively managed by the protocol might not be adopted in practice due to potential liabilities.

Literature reports different integrative approaches to overcome these limitations. Ensemble learning combines multiple ML algorithms to enhance performance and reduce over-fitting when data is scarce. Transfer learning uses pre-trained models to improve generalisation in a new task by leveraging knowledge gained from a previous one. Moreover, Informed Machine Learning represents a novel paradigm encompassing methods trained on data and prior knowledge derived from independent sources and presented through formal representation [20]. This integration aims to strike a balance between model complexity and generalisability and proved effective in various applications, particularly in the fields of physics and engineering. In the medical domain, where structured knowledge is abundant but data is often limited and noisy, the potential for successful integration is particularly high. However, specific integration strategies and frameworks tailored to the healthcare sector remain underdeveloped. Recent contributions have made significant strides in this direction but primarily focused on the integration of ML with rule-based expert systems [21, 22]. The present work seeks to expand this paradigm, proposing a more comprehensive taxonomy for integration in the medical domain, and offering general guidelines for future integration efforts in healthcare.

### Availability of medical domain knowledge

**Fig. 1:**
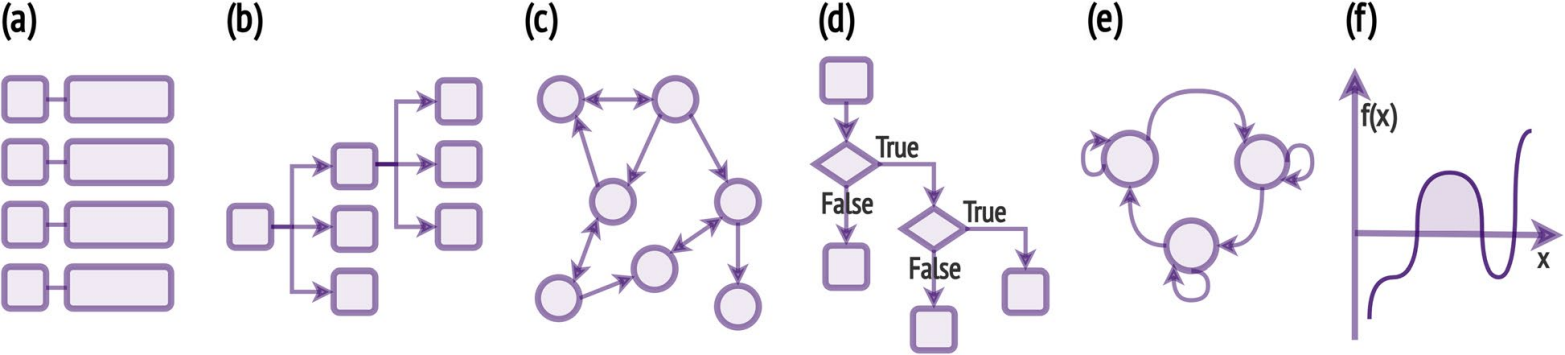
Medical domain knowledge representations: **a** vocabularies and coding systems represented as lists or **b** hierarchies of codes, **c** ontologies as knowledge graphs, **d** rules as flowcharts, **e** sequential models as Markov models, **f** functions and probability distributions

The medical field is characterised by an extensive use of terminologies. The systematic development of taxonomies, vocabularies, coding systems and ontologies reflecting a common understanding of the medical domain has enabled the representation, exchange and processing of medical knowledge. Additionally, Clinical Practice Guidelines and Care Pathways offer recommendations and best practices to support clinical decisions and guarantee consistency and continuity of care [23]. Medical and clinical domain knowledge is encoded in a variety of forms, illustrated in Fig. 1, each tailored to capture specific aspects of medical information.

- **Lists** compile diagnoses, procedures, drugs, risk factors, and genes associated with a given condition from literature reviews or expert consensus.
- **Hierarchies** classify medical codes across multiple levels. The International Classification of Diseases system is a prime example, starting with 21 broad categories of diagnoses and branching into progressively more specific sets of diagnoses.
- **Graphs** represent relationships among biological entities, such as gene co-expression networks, protein-protein interaction networks, or drug-target interactions. Knowledge graphs leverage a graph-structured data model to represent diverse medical information sourced from clinical guidelines, medical vocabularies and standards.
- **Rules** mirror clinical diagnostic reasoning and are often derived from standard clinical guidelines. Logic rules are also used to express constraints, such as anatomical constraints in medical imaging segmentation.
- **Sequential models** capture the inherent temporal order and progression in medical phenomena, modelling, for example, the steady progression of signals (e.g., heart sounds in a cardiac cycle) or pathologies (e.g., SIR models).
- **Functions** define clinical statistics and indices computed over medical data, or transformations applied to signals and images to obtain attributes with diagnostic or prognostic values. Differential equations, such as reaction-diffusion models, are employed to model complex biological systems.
- **Probability distributions** model the expectation of biological or clinical outcomes and are used in statistical inference to predict unobserved events. Threshold values are often defined to categorise continuous variables into clinically relevant intervals (e.g., low, normal, elevated).

### Previous integration of medical knowledge and ML

In recent years, there has been growing interest in integrating medical knowledge into ML models. Most efforts focused on medical data in the form of images and text, leveraging results from natural language processing and image recognition.

#### Diagnostic images and signals.

ML has proven to be a powerful tool in medical image analysis, allowing for the extraction of unbiased, low-level features predictive of clinical outcomes. Convolutional neural networks (CNNs) have been used to detect and classify anatomical abnormalities associated with diseases, with recent work integrating domain knowledge throughout all phases of the learning pipeline. This includes expert-guided rough segmentation of fundus images [24], automated filtering and cropping of MRI scans [25], and extraction of different numbers of frames in the processing of ultrasound videos to mirror the varying attention of physicians [26]. CNN-learned features have been augmented with hand-crafted features designed by experts to identify cervical lesions in liquid-based cytology [27] and red lesions on fundus images [28], and medical ontologies have been leveraged in MRI data analysis to draw meaningful feature subsets for attribute bagging [29]. Learning with regularisation has proved effective in analysing finger-tapping videos while conforming to clinical guidelines [30], in improving the segmentation accuracy of lesions while maximising consistency with anatomical knowledge in cone beam computed tomography [31, 32], and in attenuation and scatter correction in PET imaging [33]. Expert-defined rules have also been used to post-process ML model outputs in diagnostic labelling [34] and correct mistakes in the segmentation of fetal scans [35].

ML has also enabled the automated and accurate analysis of medical signals such as phonocardiogram (PCG), electrocardiogram (ECG), and electroencephalograms (EEG) in monitoring and telemonitoring scenarios, where sensors are deployed to continuously track the progression of medical conditions. The integration of domain knowledge is pivotal in the initial stages of ML pipelines, including data acquisition, preprocessing, and feature engineering. Some applications include signal denoising with expert-guided thresholds [36], signal segmentation leveraging known patterns in the cyclical nature of the heart cycle in automated PCG analysis [37], and the extraction of handcrafted features with strong physiological basis from ECG [38] and EEG [39]. In telemonitoring, where labelled data is scarce, domain knowledge was utilised to provide weak labelling in raw sensor data analysis for tapping activity [25].

#### Electronic health records

Electronic health records (EHRs) contain valuable narrative data from clinical notes, discharge summaries, and surgical records. In the analysis of free-text clinical notes, integrating domain knowledge is particularly crucial in the feature engineering phase to create effective text representations. This is especially relevant in text classification tasks like named entity recognition, relation extraction, and assertion detection, where feature engineering typically depends on dictionaries of related terms manually curated by experts. To streamline this process, tools like the Medical Language Extraction and Encoding System and KnowledgeMap [40], vocabularies from the Unified Medical Language System [41], biomedical knowledge graphs [42], and curated dictionary lookup modules [43] have been employed to automate feature engineering, resulting in one-hot vectors or embedding layers. Moreover, the hierarchical structure of diagnostic codes in the International Classification of Diseases (ICD) has been leveraged during the learning phase through refined losses [44]. Post-processing rules have also been applied to rectify potential errors in ML outputs [45]. Additionally, hybrid methods have been developed, weighting the contributions of a pattern-based method and a statistical learning method based on data availability [46].

EHRs also provide structured data, including patient demographics, laboratory tests, and medications, which can be challenging to analyse due to their high dimensionality, temporality, sparsity, irregularity and bias. To address these challenges, recent integrations have leveraged expert defined thresholds to discretise continuous variables into meaningful intervals [47], as well as previous literature [48] and existing expert models [49] to inform feature selection. Other applications have generated concise sets of meaningful summary features from expert-defined rules [50] and enriched EHR representation through the integration of knowledge graphs [51] and hierarchical code classifications [52]. Furthermore, known associations between diseases and their risk factors have been considered, either by weighting their contribution to the outcome [53] or through posterior regularisation [54]. Finally, rule-based classifiers formalising physicians’ knowledge have been combined with supervised learning algorithms by averaging ensemble [55] and voting ensemble [56].

#### Omics data

In multi-omic data analysis, a significant challenge arises from the *big p, small n* problem, where the number of features greatly exceeds the number of samples. Consequently, much of the integration effort in ML is dedicated to identifying a subset of relevant features. Biological networks [57] and medical guidelines [58] have been instrumental in guiding this selection process, ensuring that the chosen features are not only consistent with current clinical knowledge but also account for dependencies that might explain their molecular mode of action. Additionally, expert-defined rules have been employed to add virtual instances to the dataset [59]. Moreover, biological networks have been incorporated into the regularisation terms of the training objectives. This inclusion aims to minimise inconsistencies between the learned feature representation and the established feature interaction networks [60].

## Materials and methods

This section presents a taxonomy of integration strategies mapped onto the different stages of the ML pipeline, with a focus on the analysis of clinical data. This framework serves as a guideline for the implementation of hybrid ML models, here exemplified through a diabetes case study. After an overview of the dataset and existing domain knowledge, integration strategies are proposed for each phase of the ML pipeline.

### Integration pipeline

#### Data pre-processing

**Fig. 2:**
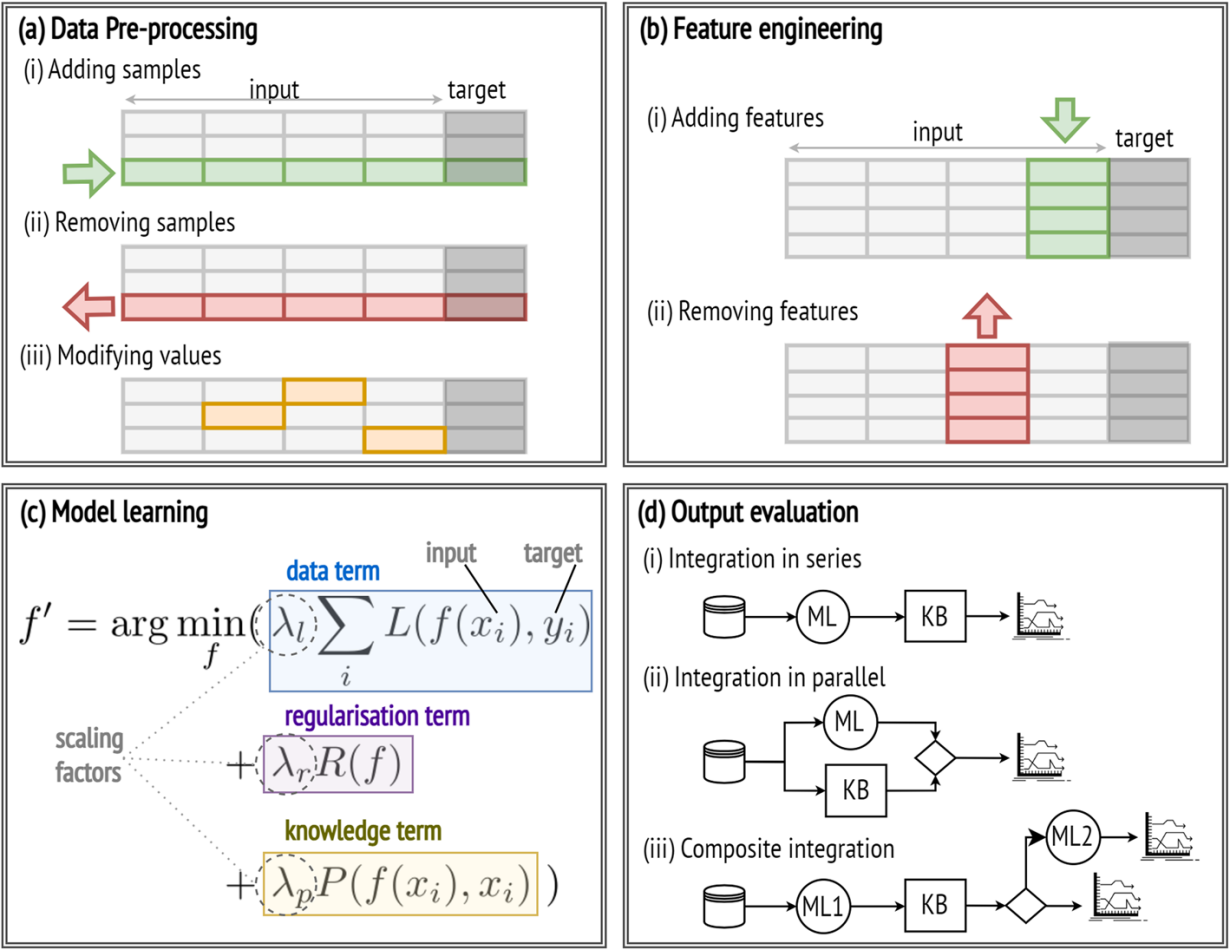
Integration strategies throughout the ML pipeline

Clinical data is often insufficient to train accurate data-driven models. To counter this, data can be supplemented by generating virtual samples that conform to the medical knowledge base’s rules and constraints, as illustrated in Fig. 2a (i), effectively mitigating data scarcity while improving the robustness and generalisation of the resulting ML models.

Clinical datasets are often marred by inconsistencies, errors, and irrelevant information due to difficulties in data collection and data reporting. In this regard, clinical norms and benchmarks can be leveraged to identify and discard data samples presenting anomalies and violations of the knowledge base constraints. This step, shown in Fig. 2a (ii), is crucial for improving the overall data quality, which in turn generally enhances the accuracy and robustness of the models.

Missing data is another frequent challenge in clinical datasets. Conventional data-driven approaches replace missing values with the mean or median, reducing dataset variability and potentially underestimating relationships among variables. However, knowledge of the underlying causal structure of the feature space, as modelled by Bayesian networks, can be leveraged to infer the most probable values for missing data based on observed variables while preserving data variability, thereby improving data quality and model accuracy.

Clinical measurements, typically recorded as continuous variables, are often interpreted by clinicians using predefined thresholds. Discretising data based on these thresholds, depicted in Fig. 2a (iii), ensures that models are trained on clinically relevant intervals. This is particularly beneficial for decision trees, which are widely used in clinical practice as they offer straightforward and interpretable models. Trees that split values according to learnt thresholds are susceptible to over-fitting and instability, as minor data variations often lead to substantial changes in the tree structure. In contrast, trees trained on discretised data are more robust and yield rules that are not only more interpretable but also more aligned with clinical knowledge.

#### Feature engineering

Clinical datasets primarily consist of features that directly measure physiological states. Before training, it is often beneficial to derive additional features from existing ones using mathematical models or logical inference from the medical knowledge base. In particular, composite indices, built upon well-established and validated medical predictive models, consolidate multiple dependent predictors into a single, clinically meaningful index predictive of disease status and treatment effects. The addition of novel features, illustrated in Fig. 2b (i), by combining several features into a few composite indices, can reduce the dataset dimensionality while enhancing model accuracy and interpretability.

The challenge of dimensionality in clinical data, characterised by numerous clinical measurements with respect to a limited number of patients, requires strategic feature selection, shown in Fig. 2b (ii). Conventional approaches evaluate linear correlations between features and the target variable and discard features with weak correlations, often overlooking the influence of confounding variables. Prior knowledge can aid in selecting relevant features by prioritising those frequently observed in the knowledge base. Additionally, known causal relationships among features, modelled by Bayesian networks, help discern confounding variables and remove redundant information. Training models on a minimal set of pertinent features mitigates the risk of over-fitting, reduces computational costs and training time, enhances interpretability, and generally improves model performance.

#### Model learning

Training ML models reduces to the optimisation problem of finding the parameter configuration for a high dimensional function that minimises the learning objective function used to evaluate a candidate solution. This function includes a term quantifying the deviation of the predicted outcomes from the ground truth of the dataset, here named the *data term*, and a *regularisation term* penalising the model coefficients to prevent over-fitting. When this function is derived solely from data, the resultant model may not always align with domain-specific constraints. To address this, prior knowledge can be incorporated by introducing an additional penalty to the loss function, referred to as the *knowledge term* [20], which quantifies any inconsistencies or violations in relation to the knowledge base, as illustrated in Fig. 2c. Incorporating this custom loss function enhances the model robustness, generalisability, and alignment with established domain knowledge.

Additionally, domain knowledge can be instrumental in shaping the model architecture. In neural networks, for instance, the network layers can be designed to pay greater attention to clinically relevant features or pathways. Similarly, in decision trees, feature selection and splitting criteria can be chosen to adhere to clinical guidelines, leading to more accurate and clinically interpretable trees.

#### Output evaluation

While ML models excel at learning complex patterns from data, traditional medical decision-making systems primarily rely on rule-based models grounded in expert knowledge. Merging these two approaches can be highly effective, leveraging the adaptability of ML models with the structured reasoning of rule-based systems, resulting in greater accuracy and alignment with established medical knowledge. Several integration architectures are possible. For instance, the predictions of an ML model can be filtered using a knowledge-based module *in series*, as in Fig. 2d (i), such that predictions that do not align with established domain rules and constraints are either discarded, flagged, or assigned a lower confidence level. Alternatively, the output of the ML model can be combined with that of a knowledge-based module (e.g. a rule-based decision system) *in parallel* so that the final outcome accounts for both predictions generated separately, as illustrated in Fig. 2d(ii). Lastly, a knowledge-based module can be used to verify the consistency of the ML prediction with domain knowledge and invoke another learning model if predictions are found to be inconsistent, as seen in Fig. 2d(iii).

### Case study

#### Dataset

The Pima Indians Diabetes Dataset is a widely used data resource in the domain of medical research, particularly in the study of diabetes. This dataset was originally compiled by the National Institute of Diabetes and Digestive and Kidney Diseases from a study of the Pima Indian population, a group with a notably high incidence of diabetes [61]. The widespread use of the dataset and the existing work on integrating ML with diabetes knowledge offer readily available domain knowledge and make it an ideal candidate for illustrating various data integration strategies. However, it should be noted that ethical concerns have emerged regarding the collection of this data [62].

The dataset comprises 768 medical profiles of women aged 21 and above, who underwent an Oral Glucose Tolerance Test (OGTT) to measure their glucose and insulin levels at two hours. The target variable is binary, indicating a diabetes diagnosis within five years. The dataset contains missing values in the attributes $I_{120}$ (48.70%), *ST* (29.56%), *BP* (4.55%), *BMI* (1.43%), and $G_{120}$ (0.65%). Dataset details are provided in Table 1.

**Table 1:** Pima Indians diabetes dataset

Feature name	Code	Description
Pregnancies		Number of times pregnant
Glucose	$G_{120}$	2-hour plasma glucose concentration in OOGT
Blood Pressure	*BP*	Diastolic blood pressure in *mmHg*
Skin Thickness	*ST*	Triceps skin-fold thickness in *mm*
Insulin	$I_{120}$	2-hour serum insulin in $\mu U/ml$
Body mass index	*BMI*	Body mass index as weight/(height)^2^ in $kg/m^2$
Diabetes Pedigree Function	*DPF*	Likelihood function of diabetes based on family history [61]
Age		Age in years

#### Domain knowledge

**Fig. 3:**
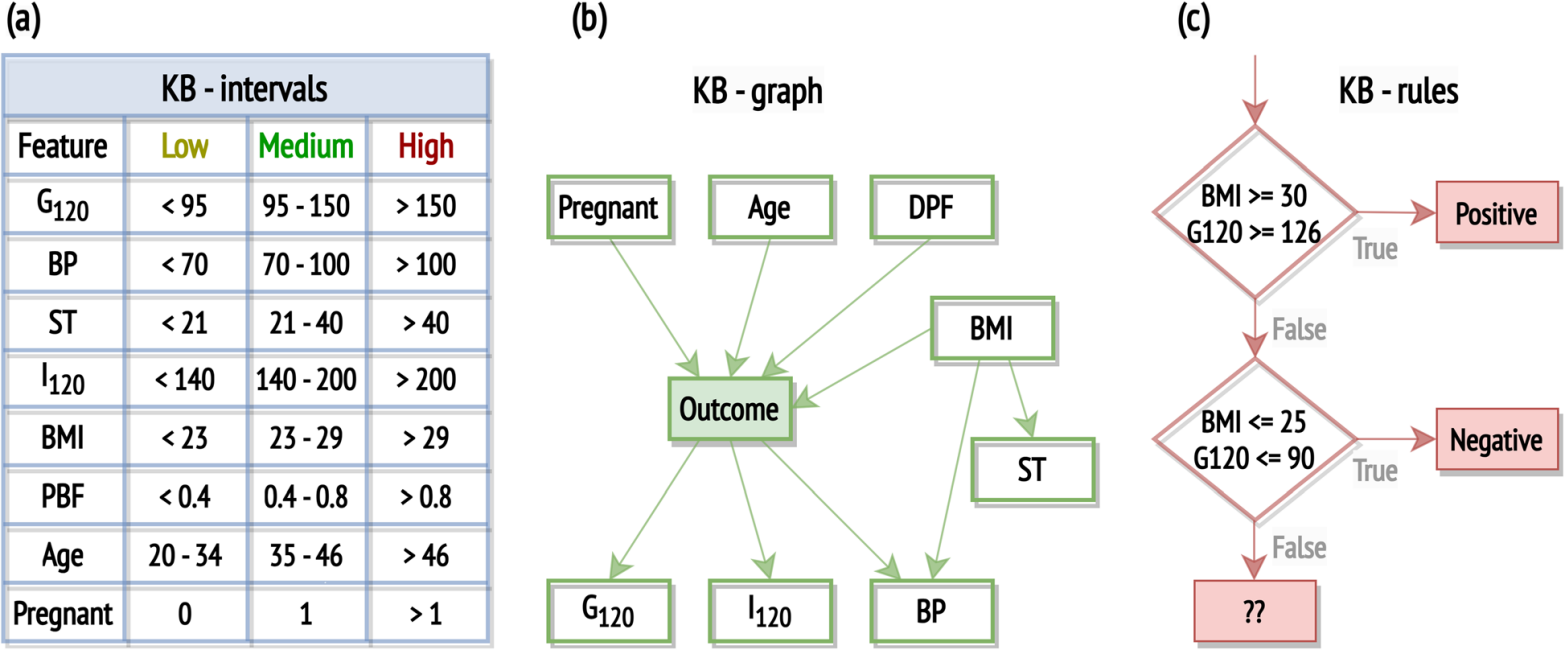
Three approaches for encoding domain knowledge in the context of the Pima Indian Diabetes Dataset, as identified in existing literature: the definition of feature intervals, with clinical interpretations for all features except *Age* and *Pregnant* [63], the construction of a Bayesian network [33], and the formulation of rules [64]

The integration of domain knowledge into ML models trained on the Pima Indians Diabetes Dataset has been the focus of various research efforts. For instance, domain knowledge was leveraged to determine realistic ranges for medical attributes. In the dataset, features such as $G_{120}$, *BP*, *ST*, $I_{120}$, and *Age* exhibit zero values, which are physiologically implausible and therefore need to be handled as missing data. Furthermore, intervals for each attribute were established based on expert knowledge as outlined in Fig. 3a, defining what test or measurement results fall into normal, abnormally high, or low ranges [63].

Previous work has also explored Bayesian networks grounded in clinical knowledge of diabetes [33]. Factors like age, family history of diabetes, pregnancy, and being overweight (estimated by *BMI*) are acknowledged as potential causes of diabetes. Skin thickness, while indicative of being overweight, has shown weaker associations with diabetes. Tests like glucose tolerance tests and serum insulin levels are direct diabetes indicators. Both obesity and diabetes are established as causative factors for elevated blood pressure. The resulting network is shown in Fig. 3b.

Public health guidelines on type-2 diabetes risks report that individuals with a high *BMI* ($\ge$ 30) and high blood glucose level ($\ge$ 126) are at severe risk for diabetes, while those with normal *BMI* ($\le$ 25) and low blood glucose level ($\le$ 100) are less likely to develop diabetes. These guidelines have been utilised to design rules [64] which can be represented as a flowchart, as shown in Fig. 3c, or expressed as logic predicates, listed in Table 2.

**Table 2:** Knowledge base for predicting risk of type-2 diabetes as formalised by Kunapuli et al. (2010) [64]

Rule 1	$(BMI \ge 30) \wedge (G_{120} \ge 126) \implies \text {diabetes}$
Rule 2	$(BMI \le 25) \wedge (G_{120} \le 100) \implies \text {healthy}$

In clinical practice, several indices have been defined to estimate insulin resistance and sensitivity. Most indices rely on both fasting glucose and insulin levels (not reported in the considered dataset), as well as measurements taken at 120 minutes during an OGTT (included in the dataset). However, one of the most common formulations of the Stumvoll index, a widely recognised formula for estimating insulin sensitivity, incorporates the 2-hour insulin measurement along with other demographic data available in the considered dataset [65]:

1 $$\begin{aligned} Stumvoll_{demographic} = 0.222 - 0.00333 \times BMI - 0.0000779 \times I_{120} - 0.000422 \times Age \end{aligned}$$

#### ML models and metrics

Missing data was imputed with the median value of the respective variable unless specified otherwise. For neural network training, data was scaled to a range between 0 and 1 with min-max normalisation.

The evaluation leverages decision trees, random forests and 3-layer neural networks, which were preferred to more complex architectures such as deep neural networks, as they lack interpretability and generally require large training sets, making their application in clinical settings often unfeasible. In contrast, simpler models are able to offer a balance between accuracy and interpretability. In particular, in this study, decision trees were chosen when the interpretability of rules was crucial, while random forests and neural networks were preferred when performance took precedence over interpretability. In particular, decision tree and random forest models were trained with a maximum depth of 10 and a minimum sample split of 5 to mitigate over-fitting. The class weight parameter was set to ’balanced’ to enhance accuracy for class one. Neural networks were implemented as a feed-forward type, consisting of three fully connected layers: two hidden layers with rectified linear unit activation functions and an output layer with a sigmoid activation function. The default loss function was the binary cross-entropy function. Neural networks were trained with a batch size of 20 for 24 epochs.

Performance was evaluated using accuracy (A), F1-score (F1), recall (R), precision (P), balanced accuracy (BA), area under the curve of the Receiver Operating Characteristic curve (ROC) and Matthews correlation coefficient (MCC) [66]. The data was divided into training and testing sets using a 10$\times$10-fold stratified cross-validation approach, which is recommended for enhancing results reproducibility [67]. The performance of each integrated model was evaluated against its corresponding data-driven model using a paired Student-t test with the Nadeau and Bengio correction [68]. The results table presents the averages and standard deviations of the performance metrics across the 100 iterations. Significance levels are denoted by *, **, and ***, indicating that the performance index of the corresponding model is significantly better than the other model at 0.1, 0.05, and 0.01 significance levels, respectively.

#### Proposed integrations

In accordance with the guidelines outlined in the Integration pipeline section, this section details the implementation of two integration strategies for each phase of the ML pipeline.

##### Data preprocessing

 

- **Continuous data discretisation**: numerical variables were converted into categorical ones using the predefined intervals informed by domain knowledge shown in Fig. 3a and then transformed into binary variables by one-hot encoding. Decision trees were trained on the original numerical dataset and the transformed categorical dataset.
- **Missing data imputation**: numerical variables were discretised according to the knowledge-based intervals in Fig. 3a and a Bayesian network structured as per Fig. 3b was trained using the Python package for Bayesian network learning and inference *bnlearn* [69]. Missing values in the training and test sets were imputed by Bayesian inference from parent variables using the Bayesian network trained on the training set. In cases where multiple outcomes were assigned the same probability, a preference was given to the median value of the feature within the dataset. If the outcomes did not include the median value, the less extreme outcome was selected, e.g. if both *Medium* and *High* were assigned equal probabilities, the *Medium* outcome would be selected. The analysis was limited to samples where the features constituting root nodes (*Pregnant*, *Age*, *BMI*, *DPF*) had no missing values (98.56% of the dataset). Random forests were trained on datasets of categorical variables with missing values imputed either using median values or Bayesian inference.

##### Feature engineering

 

- **Feature selection**: according to the Bayesian network in Fig. 3b, the *SK* feature was found to be indirectly related to diabetes through its association with obesity and excluded from the analysis. Random forest classifiers were trained on numerical datasets with and without the *SK* feature.
- **Computing novel features**: the insulin sensitivity index $Stumvoll_{demographic}$, as defined in Eq. 1, was computed and used instead of its three constituent indices, *BMI*, $I_{120}$ and *Age*. Decision trees were trained on datasets before and after incorporating this novel feature. This analysis was limited to samples where the features composing the insulin sensitivity index had no missing values (51.21% of the dataset). Feature importance analysis by feature permutation was conducted on the integrated model.

##### Model learning

 

- **Custom loss function**: a neural network was trained with a modified binary cross-entropy loss function, which assigned a higher weight to samples accurately predicted by the clinical guidelines represented by the two logic rules in Table 2. Formally, consider a training set with *N* samples and let *y*, *p*, and *r* be $N \times 1$ vectors, where *y* contains the true binary labels, *p* the output probabilities predicted by the neural network, and *r* the predictions according to the rules in Table 2. In particular, elements in *r* are assigned a value of 1 if the corresponding sample satisfies the conditions of the first rule, 0 if it satisfies the conditions of the second rule, and a null value otherwise. Then, the Custom Total Loss (CTL) for the integrated model is computed as:
  2 $$\begin{aligned} \text {CTL}(y, p, r, \alpha ) = \frac{1}{N} \sum\limits_{i=1}^{N} \text {CSL}(y_i, p_i, r_i, \alpha ) \end{aligned}$$
  where $\alpha$ is the scaling factor controlling the influence of the additional loss term, CSL is the custom binary cross-entropy loss for a single sample defined as
  3 $$\begin{aligned} \text {CSL}(y_i, p_i, r_i, \alpha ) = \left\{ \begin{array}{ll} L(y_i, p_i) &{} \text {if}\ r_i \ne y_i \\ (\alpha + 1) L(y_i, p_i) &{} \text {if}\ r_i = y_i \end{array}\right. \end{aligned}$$
  and *L* is the standard binary cross-entropy loss for a single sample
  4 $$\begin{aligned} L(y_i, p_i) = - \left[ y_i \cdot \log (p_i) + (1 - y_i) \cdot \log (1 - p_i) \right] . \end{aligned}$$
  This customised model was trained with the parameter $\alpha$ set to 3, and its performance was compared to a neural network with the same architecture but trained with a standard binary cross-entropy loss function.
- **Model architecture**: a decision tree’s structure was modified to incorporate the two domain-specific rules from Table 2 as its initial split criteria. Beyond these rules, the tree expanded as a typical decision tree. In practice, the training data was divided into three subsets: samples that satisfied the first rule, samples that satisfied the second rule, and samples that did not satisfy either. Decision trees were then trained on each of these subsets. For the second subset, no tree was trained as all samples belonged to the same class, and the output could be directly assigned. Then, test samples were split based on the two rules, and their outcomes were predicted using the corresponding decision tree. This modified tree was evaluated against a conventionally trained decision tree of the same depth.

##### Output evaluation

 

- **Ensemble learning**: a rule-based decision unit was constructed using the rules in Table 2, assigning a probability of having diabetes 1 if the conditions of the first rule apply, 0 if the conditions of the second rule apply, and 0.5 to all other cases, treated as intermediate cases. This decision unit was utilised to predict the outcomes of samples in the test set. Subsequently, a 3-layer neural network was trained on the training set using a binary cross-entropy loss function and employed to predict the outcomes of test samples. The prediction of the rule-based decision unit and the probability in output to the neural network were then combined as follows. A sample was classified as diabetic if the sum of the outputs from the two models was 1 or greater. This occurred when the rule-based model output 1, or when it output 0.5 and the neural network predicted a probability greater than 0.5, or even when the rule-based model output 0 but the neural network confidently predicted 1. This integrated approach was also tested on random subsets of 200 samples from the original dataset to evaluate its ability to handle limited data.
- **Output filtering**: a 3-layer neural network was trained on the training set using a binary cross-entropy loss function and employed to predict the test samples. Then, a rule-based decision unit was constructed based on the rules in Table 2, predicting diabetes if the conditions of the first rule applied, a healthy outcome if the conditions of the second rule applied, and providing no prediction in all other cases. Subsequently, the predictions of the neural network were cross-checked against those of the rule-based module. In cases where the rule-based module provided a prediction that differed from that of the neural network, the outcome was disregarded. This filtering impacted only those predictions that fell within the scope of the rules, and, on average, removed 5.5% of the predictions.

## Results

The performance metrics of each proposed integrated model compared to its corresponding data-driven counterpart are detailed in Table 3, classified according to the respective phases of the ML pipeline.

**Table 3:** Mean ($\bar{x}$) and standard deviation (*s*) of performance metrics computed for fully data-driven models, labelled as ML, and models incorporating domain knowledge, denoted as KB-ML, trained by 10$\times$10-fold cross-validation

		Data-preprocessing				Feature engineering				Model learning				Output evaluation			
Metric		Discretisation		Missing data		Feature Selection		Novel features		Loss function		Architecture		Ensemble learning		Output filtering	
		ML	KB-ML	ML	KB-ML	ML	KB-ML	ML	KB-ML	ML	KB-ML	ML	KB-ML	ML	KB-ML	ML	KB-ML
A	$\bar{x}$	0.712	0.688	0.713	0.720	0.762	0.770	0.721	0.719	0.764	0.754	0.713	0.695	0.765	0.759	0.767	0.779**
	*s*	(0.045)	(0.047)	(0.048)	(0.049)	(0.044)	(0.043)	(0.074)	(0.067)	(0.042)	(0.045)	(0.045)	(0.054)	(0.043)	(0.043)	(0.043)	(0.044)
BA	$\bar{x}$	0.701	0.675	0.696	0.702	0.743	0.752	0.701	0.702	0.725	0.747**	0.703	0.689	0.726	0.738*	0.729	0.747***
	*s*	(0.048)	(0.051)	(0.051)	(0.053)	(0.050)	(0.051)	(0.080)	(0.074)	(0.051)	(0.050)	(0.047)	(0.056)	(0.053)	(0.052)	(0.052)	(0.054)
P	$\bar{x}$	0.579	0.549	0.589	0.598	0.656	0.666	0.579	0.573	0.690**	0.632	0.581	0.556	0.690**	0.653	0.692	0.692
	*s*	(0.060)	(0.063)	(0.075)	(0.074)	(0.064)	(0.062)	(0.108)	(0.098)	(0.073)	(0.062)	(0.061)	(0.070)	(0.071)	(0.064)	(0.072)	(0.072)
R	$\bar{x}$	0.664	0.632	0.639	0.641	0.680	0.693	0.642	0.650	0.597	0.721***	0.668	0.670	0.598	0.668***	0.603	0.647***
	*s*	(0.087)	(0.099)	(0.095)	(0.093)	(0.093)	(0.095)	(0.132)	(0.131)	(0.099)	(0.088)	(0.083)	(0.091)	(0.103)	(0.099)	(0.097)	(0.102)
F1	$\bar{x}$	0.692	0.666	0.691	0.697	0.740	0.748	0.692	0.692	0.730	0.737	0.694	0.678	0.731	0.735	0.734	0.750**
	*s*	(0.047)	(0.049)	(0.051)	(0.052)	(0.048)	(0.048)	(0.078)	(0.072)	(0.051)	(0.048)	(0.046)	(0.055)	(0.052)	(0.049)	(0.052)	(0.051)
ROC	$\bar{x}$	0.701	0.679	0.696	0.702	0.743	0.752	0.701	0.702	0.725	0.747**	0.703	0.689	0.726	0.738*	0.729	0.747**
	*s*	(0.048)	(0.051)	(0.051)	(0.053)	(0.050)	(0.051)	(0.080)	(0.074)	(0.051)	(0.050)	(0.047)	(0.056)	(0.053)	(0.051)	(0.052)	(0.054)
MCC	$\bar{x}$	0.393	0.679	0.389	0.400	0.484	0.501	0.396	0.396	0.469	0.482	0.396	0.368	0.470	0.475	0.475	0.504***
	*s*	(0.093)	(0.098)	(0.102)	(0.105)	(0.096)	(0.096)	(0.156)	(0.145)	(0.099)	(0.097)	(0.092)	(0.109)	(0.101)	(0.098)	(0.101)	(0.102)
model		DT	DT	RF	RF	RF	RF	DT	DT	NN	NN	DT	DT	NN	NN	NN	NN
knowledge			intervals		graph		graph		formula		rules		rules		rules		rules

*ML* models encompass decision trees (*DT*), random forests (*RF*), and artificial neural networks (*NN*)

*, **, and *** indicate that the performance index of the corresponding model is significantly higher than the other model at 0.1, 0.05, and 0.01 significance levels, respectively

### Data-preprocessing

The integration strategy leveraging **continuous data discretisation** reported improved interpretability without loss of accuracy. Comparative analysis of the models trained on both the processed and original datasets revealed comparable performance metrics, with statistical evaluations showing no significant differences. Therefore, the discretisation process did not adversely affect the model predictive capabilities despite reducing the dataset information content. The ability to retain predictive power despite a loss of information suggests that the discretisation thresholds, informed by domain expertise, effectively capture intervals critical to the problem at hand. Moreover, models trained on the discretised data exhibited increased resilience to data variability, due to the fact that variations within the same categorical range do not alter the structure of the decision trees. Most importantly, these models also demonstrated improved interpretability and clinical relevance. The resulting decision rules include clinically meaningful thresholds, making them more applicable in a healthcare context. For illustrative purposes, branches from decision trees trained on the numerical and categorical datasets are shown below to exemplify how discretisation provides more interpretable and clinically meaningful decision rules.

$$\begin{aligned} \begin{array}{l} \text {Numerical dataset:}\\ (G_{120} \le 123.5) \wedge (BMI \le 30.95) \wedge (DPF \le 0.08) \wedge (Age \le 29) \wedge (Pregnant \le 7) \\ \qquad \qquad \qquad \qquad \qquad \qquad \qquad \qquad \qquad \qquad \qquad \qquad \qquad \qquad \quad \implies \lnot diabetes \end{array} \end{aligned}$$

$$\begin{aligned} \begin{array}{l} \text {Categorical dataset:}\\ (G_{120} \ne \text {High}) \wedge (BMI \ne \text {High}) \wedge (DPF \ne \text {High}) \wedge (Age \le 35) \wedge (Pregnant \le 1) \\ \qquad \qquad \qquad \qquad \qquad \qquad \qquad \qquad \qquad \qquad \qquad \qquad \qquad \qquad \quad \implies \lnot diabetes \end{array} \end{aligned}$$

In integration by **missing data imputation**, improvement was observed across all metrics, although these differences were not found to be statistically significant. Nonetheless, Bayesian inference stands out as a competitive strategy for data imputation, as it assigns more realistic values to missing data. For instance, in the dataset, three patients with high *BMI*, high *DPF*, and either high $G_{120}$ or multiple pregnancies were assigned low insulin levels via median imputation. However, Bayesian imputation predicted medium to high insulin levels, which are more plausible for this patient profile.

### Feature engineering

In the case of **feature selection**, every variable demonstrated a significant correlation with the target outcome (Pearson correlation *p*-value < 0.001) so data-driven methods cannot be effectively applied to select relevant features. However, the Bayesian network structure revealed that the feature *SK* is indirectly linked to diabetes solely through its association with obesity, while all other features directly cause or are caused by the target outcome. Models trained on datasets both with and without the *SK* feature yielded similar performance, suggesting that *SK* does not contribute essential information to the model. Feature exclusion strategies informed by domain knowledge are expected to prove more beneficial in datasets with a larger number of features, where only a few are predictive, as opposed to the present dataset which contains a limited and already curated set of features.

Similarly, in the integration strategy **computing novel features**, models trained on a dataset where the composite index $Stumvoll_{demographic}$ is used in place of its three constituent features reported performance comparable to those trained on the original dataset, with no statistically significant differences. Notably, feature importance analysis highlighted $Stumvoll_{demographic}$ as the second most relevant feature following $G_{120}$, underscoring the robust predictive value of this composite index. Furthermore, including an indicator of insulin sensitivity in the model can enhance its interpretability and contribute to more clinically relevant insights.

### Model learning

The implementation of a **custom loss function** significantly enhanced both performance and adherence to established clinical guidelines. This tailored approach led to a significant increase in recall, a critical measure in diagnostic contexts, from 0.597 to 0.721. Furthermore, in evaluating the samples correctly handled by the clinical guidelines, the integrated models predicted these samples with 97% accuracy, significantly higher than models trained with the standard loss function with only 90% accuracy (*p*-value = 0.00003). Importantly, both ML models demonstrated predictive capabilities exceeding those of the clinical guidelines, which only cover 35% of samples and correctly predict 26%. However, the model trained with the custom loss function achieved a higher rate of diabetes detection and near-complete adherence to existing guidelines, increasing the likelihood of its practical adoption in clinical settings.

Integration by altering the **model architecture** yielded performance comparable to fully data-driven trees. The modified decision tree categorises data into three clinically meaningful groups: samples likely to have diabetes as per rule 1, samples unlikely to have it according to rule 2, and a third intermediate category. This initial segmentation not only aligns with clinical insights but also enhances the overall interpretability of the tree structure. It provides a more nuanced characterisation of each group, potentially guiding more tailored care guidelines.

### Output evaluation

In the context of **ensemble learning**, the integrated model combining the outputs of an ML module and a rule-based unit reported a significant improvement in recall with respect to the ML module alone. Notably, due to the stringent criteria combining these outputs, the integrated model accurately predicted all samples correctly handled by the protocol. This threshold can be adjusted to modulate the adherence to the rules: a threshold above 1 allows ML to only affect the decision on intermediate values, which the rule module cannot evaluate, while a lower threshold enables the ML module to overthrow the decision of the rule module. In the data efficiency analysis, the ensemble model maintained stable overall performance, demonstrating its robustness against reduced data availability, while the performance of the neural network alone experienced a significant drop across all performance metrics.

Finally, the **output filtering** integration strategy also proved effective in providing more accurate predictions, with a significant improvement in all performance metrics except precision. Moreover, this approach guarantees adherence to the rules by design, as it discards all predictions contradicting the rule-based filter.

## Discussion

The comprehensive literature review and the diabetes case study collectively attest to the efficacy of incorporating domain knowledge across diverse phases of the ML pipeline, ranging from data preprocessing to output evaluation, in clinical settings. This integration holds tremendous promise in improving ML models in terms of accuracy, data efficiency, interpretability and coherence with established clinical protocols, addressing critical challenges inherent to building clinical predictive models. Overall, integration efforts also increase the transferability of the models and relevance in real-world medical settings, streamlining their deployment within clinical practice. Nonetheless, this process is not without its challenges, such as knowledge representation and integration fine-tuning, which require further investigation.

### Accuracy

Integrating medical domain knowledge throughout the entire ML pipeline, starting from the initial stages of data cleaning and preprocessing, holds the potential to improve model performance [59]. Multi-dimensional data analysis, harnessing complementary evidence from diverse data sources, is another scenario where purely data-driven strategies are often ineffective, and integrating knowledge of the underlying feature structure has proved crucial in improving model performance [26, 70].

Integration also plays a pivotal role in feature engineering, addressing the limitations of both manual and fully automated methods. Manual feature extraction, while precise, tends to be costly, time-consuming, and varies with the practitioner’s expertise [25, 39, 71]. Conversely, fully automated processes can yield low-quality results, as seen for low-frequency words in word embeddings [40]. Automated approaches grounded in domain knowledge have been adopted to ensure consistency, reduce subjectivity, and improve standardisation, ultimately leading to higher classification performance and reliability. These integrations are most relevant in the analysis of unstructured data (as in the form of images or text) where features need to be extracted, or where thousands of features are available and few relevant ones need to be selected (as in omics data analysis). In contrast, they are not as effective in improving model accuracy in structured datasets of few curated features, as seen in the proposed case study.

Integration strategies are also instrumental in improving model generalisability and mitigating the risk of over-fitting, generally acting at the level of model learning and output post-processing. This is clearly exemplified in the presented case study, where integrated strategies encompassing these two ML phases showed marked performance improvements, particularly in terms of recall. However, such approaches are expected to be even more effective in scenarios characterised by high dimensionality and small sample sizes, where purely data-driven ML models face challenges [60, 72].

### Data efficiency

Leveraging medical knowledge can mitigate the challenges posed by limited training data by constructing simpler and more robust models or ensembles of models informed by domain knowledge and trained on smaller datasets [73], as opposed to single, complex, data-intensive models. This approach was demonstrated in the presented case study, where an ensemble of two learners, a rule-based module and a neural network proved more effective than the neural network alone when reducing the training size.

In data-scarce contexts, such as clinical settings, the benefit of integration extends beyond the design of data-efficient models and has proven effective in data labelling for semi-supervised learning. While good-quality labelled data are rare or very expensive to obtain, there is generally an abundance of unlabelled data, particularly in the rising sector of telemonitoring [28, 36]. In such scenarios, domain knowledge can be leveraged to provide (weak) labelling of data and enable the use of data-intensive models [74].

### Coherence

Integration strategies can align ML models with existing medical knowledge and clinical practices, a critical aspect in fostering trust among physicians who rely on the efficacy of these long-standing guidelines. This alignment is also crucial in ensuring continuity of care and promoting the adoption of such models in clinical settings [47]. In the presented case study, integrations in learning and output evaluation phases attained near-complete or complete adherence to the guidelines while improving performance, thereby enhancing the model’s potential for clinical adoption.

Additionally, integration techniques have been used to enforce constraints derived from expert models [75, 76], particularly in image segmentation tasks [77], thus preventing models from producing outcomes that are biologically or physically implausible. Further integrations accounted for latent relationships and dependencies among features [29, 57] so that models do not make predictions based on confounding variables, improper relationships or latent variables with no physical interpretation.

### Interpretability

Embedding domain knowledge within ML models can add a layer of interpretability to the model [78] by providing the rationale behind the model’s recommendations [79] and allowing the re-traceability of the model decisions to specific model components [42]. Overall, these integration efforts also increase the transferability of the models and relevance in real-world medical settings [80, 81], streamlining their integration into clinical practice. For these reasons, recent advancements in clinical AI focused on developing simpler, more interpretable models made of a minimal set of knowledge-driven features able to provide good explanations [50, 73].

In the diabetes case study, interpretability was greatly enhanced by integrating knowledge at various stages of the pipeline. For instance, discretising data according to clinically meaningful intervals led to the generation of more relevant decision rules. Including derived composite indices enabled the model to account for complex but well-characterised physiological mechanisms like insulin resistance and sensitivity. Furthermore, grounding the model architecture on rules formalising a clinical protocol provided a nuanced characterisation of clinically relevant patient groups.

### Knowledge representation

A major obstacle hindering the broad adoption of integrative approaches concerns the formal representation of medical knowledge. The absence of a unified knowledge base results in redundancy and conflict in medical terminologies, potentially causing labelling errors and undermining the performance of ML models [82]. Furthermore, the inadequate encoding of clinical guidelines, often embedded in lengthy and complex text documents, greatly impedes their use in ML. To facilitate effective integration, there is a pressing need to address conflicts in medical terminologies and transcribe protocols into a concise, machine-readable format [83]. In the presented case study, several conflicting clinical guidelines were available for diabetes diagnosis, most of which in free-text form. The choice fell on a protocol that had been formalised into logical rules in earlier research, despite it not being the latest or most comprehensive protocol available. The selection of one set of guidelines over another likely had a considerable impact on the model outcomes.

The extensive volume of medical knowledge, encompassing over 140,000 codes in the ICD-10 taxonomies, calls for methods to navigate this vast corpus of information [44]. Medical knowledge also comes with heterogeneity of sources, from knowledge graphs to medical Q &A databases, and despite recent advancements in integrating these diverse sources, there is still considerable room for improvement [84]. A final consideration is the varying availability of domain knowledge across different medical conditions, which is abundant for common diseases but often limited for rare genetic conditions. This disparity can greatly affect the applicability of integration frameworks, which are only as effective as the extent of available knowledge [54].

### Integration tuning

Domain knowledge integration entails balancing two distinct contributions: the first based on previously established and formalised information and the second expressed as statistical and probabilistic relationships. The need for fine-tuning these contributions was evident in the presented case study, where arbitrary weights and thresholds were adopted in the custom loss function and learner ensemble, respectively. Therefore, optimising and automating this fine-tuning process stands as a critical challenge in integration, warranting further investigation [20].

## Conclusion

The article emphasises the potential of ML in clinical medicine, while also highlighting the limitations of purely data-driven approaches in capturing the complexities of medical data. Through a comprehensive review and a case study on diabetes prediction, the study illustrates the benefits of integrating medical domain knowledge into ML models across various stages of the ML pipeline. The findings reveal that integration enhances model accuracy, particularly in terms of generalisation and performance in data-limited scenarios, as well as interpretability and alignment with clinical guidelines. The selected case study, characterised by a relatively simple dataset comprising eight features and two domain knowledge rules, provided a prime opportunity to showcase the potential and feasibility of integration even in such simplified settings. Further research endeavours will be essential to investigate integration opportunities into more complex clinical scenarios involving additional data dimensions and a wider spectrum of domain knowledge rules. Finally, the study not only serves as a guide for future integration efforts but also points to the need for further research in refining the representation of medical knowledge and fine-tuning its integration into ML models.

## Data Availability

The dataset analysed in the current study is available in the Pima Indians Diabetes Database repository (https://www.kaggle.com/datasets/uciml/pima-indians-diabetes-database). Additionally, the code necessary to replicate all experiments conducted in the manuscript can be found in the corresponding GitHub repository (https://github.com/ChristelSirocchi/medical-informed-ML).

## Abbreviations

ML: Machine learning
AI: Artificial intelligence
FDA: Food and drug administration
XAI: Explainable artificial intelligence
CNN: Convolutional neural network
MRI: Magnetic resonance imaging
PET: Positron emission tomography
PCG: Phonocardiogram
ECG: Electrocardiogram
EEG: Electroencephalogram
EHR: Electronic health record
ICD: International classification of diseases
SIR: Susceptible, infectious, or recovered
OGTT: Oral glucose tolerance test
BMI: Body mass index
DPF: Diabetes pedigree function
SK: Skin thickness
BP: Blood pressure
CTL: Custom total loss
A: Accuracy
BA: Balanced accuracy
R: Recall
P: Precision
ROC: Receiver operating characteristic
MCC: Matthews correlation coefficient
CSL: Custom single loss
